# Economic benefits of the Mediterranean-style diet consumption in Canada and the United States

**DOI:** 10.3402/fnr.v59.27541

**Published:** 2015-06-24

**Authors:** Mohammad M.H. Abdullah, Jason P.H. Jones, Peter J.H. Jones

**Affiliations:** 1 Department of Human Nutritional Sciences, University of Manitoba, Winnipeg, MB, Canada; 2 Richardson Centre for Functional Foods and Nutraceuticals (RCFFN), University of Manitoba, Winnipeg, MB, Canada; 3 Department of Agricultural Economics, Texas A&M University, College Station, TX, USA

**Keywords:** cardiovascular disease, public health, healthy eating, nutrition economics, cost-of-illness analysis

## Abstract

**Background:**

The Mediterranean-style diet (MedDiet) is an established healthy-eating behavior that has consistently been shown to favorably impact cardiovascular health, thus likely improving quality of life and reducing costs associated with cardiovascular disease (CVD). Data on the economic benefits of MedDiet intakes are, however, scarce.

**Objective:**

The objective of this study was to estimate the annual healthcare and societal cost savings that would accrue to the Canadian and American public, independently, as a result of a reduction in the incidence of CVD following adherence to a MedDiet.

**Design:**

A variation in cost-of-illness analysis entailing three stages of estimations was developed to 1) identify the proportion of individuals who are likely to adopt a MedDiet in North America, 2) assess the impact of the MedDiet intake on CVD incidence reduction, and 3) impute the potential savings in costs associated with healthcare and productivity following the estimated CVD reduction. To account for the uncertainty factor, a sensitivity analysis of four scenarios, including ideal, optimistic, pessimistic, and very-pessimistic assumptions, was implemented within each of these stages.

**Results:**

Significant improvements in CVD-related costs were evident with varying MedDiet adoption and CVD reduction rates. Specifically, CAD $41.9 million to 2.5 billion in Canada and US $1.0–62.8 billion in the United States were estimated to accrue as total annual savings in economic costs, given the ‘very-pessimistic’ through ‘ideal’ scenarios.

**Conclusions:**

Closer adherence to dietary behaviors that are consistent with the principles of the MedDiet is expected to contribute to a reduction in the monetary burdens of CVD in Canada, the United States, and possibly other parts of the world.

The recent emergence of ‘nutrition economics’, the study of eating behaviors in the context of health economics, has offered better understanding of absolute and relative monetary impacts of health responses to dietary habits (1, 2). Dramatic rates in prevalence of cardiovascular diseases (CVDs), including coronary heart disease (CHD) and stroke, currently place great burdens on national economies worldwide. Globally, over 17 million, or 30%, of all deaths were attributed to CVD in 2008 (3) and costing US $863 billion in 2010 (4). Specific to North America, CVD accounted for 29% of all deaths in Canada in 2008 (5) and have been estimated to cost nearly CAD $21 billion in annual healthcare costs based on 2005 monetary figures (6). Also, about 600,000 Americans died of CHD alone in 2010 (7), with the direct and the indirect costs of the disease amounting to US $108.9 billion (8).

Although the genetic predispositions cannot be ignored, it is well-acknowledged that lifestyle changes play the largest role within strategies to prevent, delay, or manage the progression of CVD, where healthy dietary patterns sit at the heart of the scenario (9). Outcomes of the presently largest ‘hard’ endpoint dietary intervention trial PREDIMED (10) recently confirmed a wealth of knowledge regarding the cardio-protective impacts of the largely plant-centered Mediterranean-style diet (MedDiet) (11). Specifically, 30 and 40% reductions in CHD and stroke, respectively, were evident with closer adherence to a MedDiet, in comparison to a low-fat control diet. In agreement, the Lyon Diet Heart Study, a secondary prevention trial, previously showed a 50% reduction in major cardiac events in CVD patients who followed a MedDiet (12). First proposed by Ancel Keys in the 1960s (13), the classical MedDiet is characterized by high intakes of fruit and vegetables, whole grains, legumes, and tree nuts; moderate intakes of cheese and yogurt, fish, poultry, and eggs; and low intakes of red and processed meat (14). This most widely accepted model of healthy nutritional patterns (15) also emphasizes extra-virgin olive oil (EVOO) as the principal source of daily fat intake and a glass of wine almost always during supper hours (14).

In spite of the considerable cardio-protective evidence from a myriad of epidemiological and intervention studies (16–22), the healthcare and societal benefits of the MedDiet consumption have yet to be fully monetized. The objective of this research was thus to estimate the potential annual savings in CVD-related economic costs in Canada and the United States, independently, under a MedDiet using multiple scenarios of intake.

## Methods

**Fig. 1:**
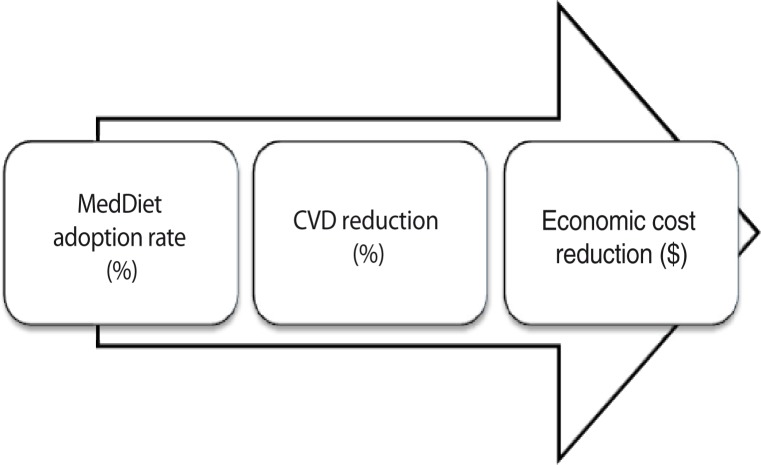
The study's economic framework utilizing a variation of cost-of-illness analysis of three stages of estimations. Based on data from recent peer-reviewed literature and national databases, the first stage identified the proportions of individuals who are likely to adhere to a MedDiet in Canada and the United States, the second assessed the reported cardiovascular disease reduction rate following a MedDiet consumption, and the third stage imputed the potential reduction in economic costs associated with the estimated CVD incidence reduction. In covering a wide range of predictions, each stage constituted four scenarios of assumptions reflecting best- through worst-case scenarios as follows: ideal, optimistic, pessimistic, and very-pessimistic.

In a conceptual framework design, a three-stage variation of cost-of-illness analysis was employed. This is an adapted version of a model that was originally presented by Malla et al. (23) and Gyles et al. (24) for economic benefits of canola oil and plant sterol consumptions, respectively. The first stage of the present model identified the proportion of individuals who are likely to adhere to a MedDiet in Canada and the United States based on the available nutrition literature. The second stage, again based on current medical literature, assessed the action of a MedDiet in terms of CVD incidence reduction. Finally, the third stage imputed the potential savings in healthcare- and society-related costs following the estimated CVD risk reduction. Figure 1 depicts the three stages of the economic model described in this article. In quest of the most robust predictions in a range of assumptions, four scenarios were generated within each stage of assessment using a sensitivity analysis as follows: ideal (best-case scenario), optimistic, pessimistic, and very pessimistic (worst-case scenario), as previously (24).

### Stage 1: Assessment of the Mediterranean-style diet intake, the ‘adoption rate’

The adoption rate refers to the proportion of individuals who are likely to adhere to a MedDiet. In predicting the extent to which Canadians and Americans may be able to adopt this particular dietary pattern, presumably leading to improvements in cardiovascular health, and thus healthcare cost savings, an extrapolation of MedDiet score (MedScore) data of North American studies in the literature was utilized. Diverse in design, the MedScores are questionnaire-like scoring systems designed to assess the habitual or recommended degrees of adherence to a MedDiet; some MedScores have been validated in different populations (25). Data from studies employing MedScores reveal adoption rates that range between 5 and 65% (26–35).

### Stage 2: Evaluation of the reduction in incidence of CVDs following intakes of the Mediterranean-style diet

A myriad of cohort and randomized controlled studies with a wide spectrum of constructs have documented beneficial impacts of the MedDiet on CVD risk and/or mortality, with protection rates ranging between 10 and 70% (10, 12, 30, 32, 33, 36–41). Akin to Stage 1 of this analysis, potential rates of reduction in incidence of CVD following intakes of the MedDiet were established based on data in the literature. Therefore, we focused on evidence deriving, as appropriate, from original and well-designed primary or secondary prevention trials that had involved male and female participants, investigated cardiovascular impacts of the MedDiet in its entirety (not merely single foods or nutrients of it), and reported adjusted relative risks (RR), hazard ratios (HR), or odds ratios (OR) for CVD with corresponding 95% confidence intervals. Data for this stage of the analysis were derived from studies conducted both in North America and Europe.

### Stage 3: Calculation of the potential savings in CVD-related economic costs

In the third and final stage of this analysis, recent estimates of economic costs associated with CVD in Canada (42, 43) and the United States (44) were initially inflated to their 2014 monetary equivalents, and then separately employed in the calculations of potential cost savings in the two countries under study. The point of view for this cost analysis is of particular importance due to the vastly different healthcare system funding schemes between Canada and the United States. The analysis provides estimates of direct cost savings from a provider prospective and an indirect saving component based on costs faced by the society. While the analysis provides critical components for a societal analysis, a complete evaluation from a societal viewpoint would require an economic evaluation of consumer choice and food market consequences. Drummond et al. (45) detail these various assessment perspectives and recommend appropriate methodologies for evaluating various healthcare programs. These methodologies are transferable to the current research for assessing the healthcare cost impact of reduced disease prevalence, where the authors provide generalized approaches for evaluating shared, direct, and indirect associated costs.

### Economic costs of CVDs in Canada

Recent data on the economic costs attributed to CVD rates within Canada were extracted from the *Economic Burden of Illness in Canada (EBIC) 2005–2008* report (42) and the *National Health Expenditure Trends (NHEX) 1975–2013* report (43). Statistics Canada's Health Care Consumer Price Index (CPI) was subsequently used to translate the costs of CAD $12,188.8 million in 2008 into the 2014 dollars of 13,042.1 million (Table 1). As presented by *EBIC 2005–2008* and summarized in Table 1, the CVD-related economic costs in Canada were divided into direct and indirect categories. The former represents costs associated with hospital operations, medications, and physicians’ and other healthcare professionals’ payments, whereas the latter deals with cost estimates that relate to reduced productivity due to mortality, illness, and disabilities. Seeking closer estimations on cost savings that would accompany each 1% reduction in CVD incidence, components of the direct and indirect categories were assessed individually, similar to previously (24).

**Table 1:** Summary of the economic costs attributed to CVD in Canada (CAD $million)

Year		2008^a^	2014^b^
Direct costs	Hospitals	5,068.0	5,422.8
	Drugs	4,272.7	4,571.8
	Physicians	2,352.0	2,516.7
	Other directs^c^	134.1	143.5
	Total direct	11,826.8	12,654.7
Indirect costs	Mortality	92.4	98.9
	Morbidity	269.6	288.5
	Total indirect	362.0	387.3
Total CVD costs		12,188.8	13,042.1

a From the *Economic Burden of Illness in Canada (EBIC) 2005–2008* report (42).

b Current dollars based on adjustments according to Statistics Canada's Health Care Consumer Price Index.

c Comprises costs for ‘Other Professionals’ (chiropractors, physiotherapists, private duty nurses, etc.) and ‘Other Health Spending’ (home care, medical transportation, etc.) by the *National Health Expenditure Trends (NHEX) 1975–2013* report (43), where the total ‘Other Direct’ costs for all diseases in 2008 equaled CAD $53,022.1 million. Percentage of CVD relative to total ‘Other Direct’ costs within the *EBIC 1998* report (46) of 0.3% was used to estimate our 2008's ‘Other directs’ monetary figure of CAD $134.1 million.

A 1999 cost analysis study (47) suggested that 84% of hospitalization costs are ‘fixed’ (i.e. salaried labor, buildings, and equipment), thus not affected by the number of disease cases, whereas 16% are ‘variable’ (i.e. medications and supplies). Our economic model thus presumed that each 1% reduction in the incidence of CVD would logically be followed by a 0.16% reduction in hospital costs. Notably, this projection is conservative when compared to the 25–42% variable hospitalization cost figure previously reported by other authors (48, 49). Given that fewer CVD cases would, as well, sensibly lead to fewer drugs prescribed for treatment, it was presumed that each 1% reduction in CVD incidence would be accompanied by a comparative 1% reduction in cost of medications. The same concept was applied to the physician care component of the direct cost category, where fewer visits to physician offices would in theory result in lower billing-related costs; therefore, a 1% lower CVD rate was assumed to lower this category also by 1%. The last component of the direct cost category, the ‘other directs’, comprises costs for other institutions, other professionals (dental services, vision care services, and other), capital, public health, and other health spending (e.g. health research) (42). Some of these medical services were logically assumed not to constitute a significant part of the CVD-related cost burden and were hence not included in our valuation. The other direct health expenditure totals were included in the *EBIC 2005–2008* report; however, they were not attributed by EBIC categories, such as certain sex, age group, or a particular diagnosis. Our model thus relied on data from the *NHEX 1975–2013* (43) report to assume that a 1% decrease in the incidence of CVD would translate into a 0.19% decrease in costs of the ‘other directs’ component. This value was based on estimations of services by the *NHEX 1975–2013* ‘Other’ (5.4%), within the ‘Other Professionals’ category, defined as costs for chiropractors, physiotherapists, private duty nurses, and others, as well as services by the ‘Other’ (13.8%) within the ‘Other Health Spending’ category, defined as costs for home care, medical transportation, training of health workers, and others. Finally, costs that are associated with the two components of the indirect category were, on the other hand, predicted to decline in proportion to the CVD incidence reduction, based on the assumption that as the number of individuals with CVD decreases, a reduction in the premature mortality and disability rates would follow proportionately (Table 2).

**Table 2:** Summary of the cost reductions corresponding to 1.0% reduction in incidence of CVD within Canada and the United States

	Cost reduction (%)			
	
	Canada		United States	
Direct reduction	Hospitals	0.16	Hospital, inpatient^b^	0.16
	Drugs	1.00	Hospital, emergency^c^	0.16
	Physicians	1.00	Hospital, outpatient^d^	0.16
	Other directs^a^	0.19	Home healthcare	1.00
			Prescribed medicines	1.00
Indirect reduction	Mortality	1.00	Lost productivity/mortality	1.00
	Morbidity	1.00		

All hospital figures are based on the estimation that 16% of hospitalization costs are variable (i.e. medications and supplies) and 84% are fixed (i.e. salaries, buildings, and equipments) (47).

a Based on estimations of services by the *National Health Expenditure Trends (NHEX) 1975–2013* ‘Other’ (5.4%), within the ‘Other Professionals’ category and ‘Other’ (13.8%) within the ‘Other Health Spending’ category (43).

b Refers to room and board, and all diagnostic and laboratory costs.

c Refers to diagnostic and laboratory costs.

d Includes office-based provider visits, laboratory costs, and visits to medical providers. The US categories are from Go et al. (44).

### Economic costs of CVDs in the United States

Calculations of the potential savings in economic costs associated with CVD for the United States were conducted in a manner similar to that of Canada, but using separate data sets. Go et al. (44) published a report on behalf of the American Heart Association (AHA), incorporating the Medical Expenditure Panel Survey data with current demographic figures to produce the direct (hospital, home healthcare, and prescribed medicines) and indirect (lost productivity/mortality) costs associated with CVD rates in the United States. There, increased detail on hospital costs (i.e. inpatient, emergency, and outpatient) was available, with physician costs included in the ‘Hospital outpatient or office-based provider visits’ category. We used monetary adjustments according to the US Department of Labor's Health Care CPI to translate the CVD-associated costs of US $315.4 billion in 2010 into $346.9 billion in 2014 (Table 3). Similar to the Canadian context, the corresponding percentage decreases from a 1% reduction in CVD were estimated individually. All hospital-related costs were assumed to only decrease by their respective approximated 0.16% ‘variable’ costs, as discussed earlier. Home healthcare is in theory dependent on disease prevalence; thus, a 1:1 relationship between CVD prevalence and costs was assumed, which was also the case with medications as well as the only indirect cost component, ‘lost productivity/mortality’ (Table 2).

**Table 3:** Summary of the economic costs attributed to CVD in the United States (US $billion)

Year		2010^a^	2014^b^
Direct costs	Hospital, inpatient^c^	98.0	107.3
	Hospital, emergency^d^	8.7	9.6
	Hospital, outpatient^e^	42.2	46.4
	Home healthcare	11.7	12.9
	Prescribed medicines	32.8	36.1
	Total direct	193.4	212.7
Indirect costs	Lost productivity/mortality	122.0	134.2
Total CVD costs		315.4	346.9

a Adapted from Go et al. (44, Table 24-1).

b Current dollars based on monetary adjustments according to the healthcare category of the US Department of Labor's Health Care Consumer Price Index.

c Refers to room and board, and all diagnostic and laboratory costs.

d Refers to diagnostic and laboratory costs.

e Includes office-based provider visits, laboratory costs, and visits to medical providers.

## Results

### Mediterranean-style diet adoption rate

Of the epidemiological and dietary intervention studies that have revealed varying levels of adherence to the MedDiet in Mediterranean and non-Mediterranean regions (26–35), MedScore findings (% of lowest and highest self-reported intakes) specific to three North American studies were of particular interest (26, 31, 32). A 12-week nutrition intervention advocating intakes of MedDiet in a ‘real life’ situation observed adoption rates of 48 and 65.5% in 73 French-Canadian women at baseline and endpoint, respectively (26). This study employed a global MedScore based on the Mediterranean diet pyramid (Oldways Preservation and Exchange Trust) of 11 components, including grains, fruits, vegetables, legumes, nuts and seeds, olive oil, dairy products, fish, poultry, eggs, and sweets and red meat/processed meat to evaluate the adherence to the recommended dietary pattern based on group and individual sessions. In the NHANES III study, involving 13,197 individuals in the United States, highest MedDiet adoption rates were reported by 27.9 and 39.5% of <45- and ≥45-year-old males, and 32.2 and 40.0% of pre- and postmenopausal females, respectively (31). In the same study, 37.5 and 30.0% of <45- and ≥45-year-old males, and 35.7 and 30.7% of pre- and postmenopausal females, respectively, reported lowest adoption rates. This study employed the scoring methodology by Panagiotakos et al. (50) of 11 components, including non-refined cereals and products, potatoes, fruits, vegetables, legumes, fish, red meat and products, poultry, full-fat dairy products, olive oil, and alcoholic beverages. Finally, in the NOMAS cohort (32), 25 and 14% of the 2,568 participants, respectively, scored highest and lowest on the employed MedDiet scoring methodology by Trichopoulou et al. (27) of nine components, including dairy, meat, fruit, vegetables (excluding potatoes), legumes, cereals (all cereals including refined and whole grain), fish, alcohol, and monounsaturated to saturated fatty acid (MUFA/SFA) ratio (32). Other North American studies reported similar adoption rates of 14–56% (51–53). In establishing MedDiet adoption rates in our sensitivity analysis that would reflect realistic ranges of intakes in a North American state, conservative rates of 50, 25, 15, and 5% were chosen to, respectively, reflect the potential ideal, optimistic, pessimistic, and very-pessimistic scenarios (Table 4).

**Table 4:** Summary of the sensitivity analysis (%)

	Scenario			
	
	Ideal	Optimistic	Pessimistic	Very pessimistic
Adoption rate	50.0	25.0	15.0	5.0
CVD incidence reduction	60.0	30.0	20.0	10.0

CVD, cardiovascular disease.

### CVD reduction

In determining the ideal scenario of CVD incidence reduction with intakes of the MedDiet based on previous research (10, 12, 30, 32, 33, 36–41), our economic model considered the 60% reduction observed in participants with the highest MedDiet adherence in the Spanish SUN cohort, which followed 13,609 individuals over 4.9 years (33). An optimistic 30% primary endpoint event reduction was chosen based on data of the group assigned to a MedDiet with EVOO in the PREDIMED trial (10). This was consistent with the 33% decreased risk rate observed in a cluster of four markers for CVD (hypertension, diabetes, obesity, and hypercholesterolemia) among individuals who reported higher MedDiet intakes in the cross-sectional data of PREDIMED, involving 3,204 Spanish participants (30). A pessimistic incidence reduction assumption of 20% was considered based on the 12-year follow-up data from the Dutch contribution to the EPIC cohort, which involved 40,011 participants (41). This was similar to the 15% lower risk previously reported with the MedDiet group of the French Medi-RIVAGE study, a 3-month parallel design dietary intervention involving 212 participants (38). Data from two cohorts in the United States (32, 40) showed similar protection rates of 20–22% and fell well within our model assumptions for the optimistic and pessimistic scenarios. Finally, a very-pessimistic CVD risk reduction scenario of 10% was chosen based on the findings from the CARDIO2000 multicenter, case–control study that involved 418 CVD patients and 303 hypertensive controls in Greece (36). The majority of these studies estimated the MedDiet consumption based on the scoring methodology by Trichopoulou et al. (27) as described above. A summary of the CVD reduction rates for Canada and the United States is shown in Table 4.

### CVD-related economic cost reduction

Our sensitivity analysis predicted total annual economic cost savings in Canada that ranged between CAD $2,511.3 and $41.9 million for the ideal through very-pessimistic scenarios (Table 5). Specifically, while the direct cost savings ranged between CAD $2,395.1 and $39.9 million, the indirect cost savings accounted for CAD $116.2 to $1.9 million annually. These estimates reflect the most recent comprehensive burden of illness data available in Canada, in conjunction with a thorough analysis of the peer-reviewed literature concerning the MedDiet and CVD incidence relationship. The wide range of economic cost savings can be primarily attributed to the variability in the latter parameter, given the consideration of such a large number of studies to determine this relationship.

**Table 5:** Potential cost savings attributed to the reduction in CVD with varying adoption rates of MedDiet in Canada (CAD $million)

	Scenario^a^			
	
	Ideal	Optimistic	Pessimistic	Very pessimistic
Direct savings				
Hospitals	260.3	65.1	26.0	4.3
Medications	1,371.5	342.9	137.2	22.9
Physicians	755.0	188.7	75.5	12.6
Other directs	8.3	2.1	0.8	0.1
Total direct	2,395.1	598.8	239.5	39.9
Indirect savings				
Mortality	29.7	7.4	3.0	0.5
Morbidity	86.5	21.6	8.7	1.4
Total indirect	116.2	29.1	11.6	1.9
Total savings	2,511.3	627.8	251.1	41.9

a The ideal scenario represents a best-case estimate of potential economic cost savings when 50% of the population follows a MedDiet and shows 60% reduction in cardiovascular disease (CVD). The optimistic scenario is a medium- to short-term pragmatic estimate of potential cost savings when 25% of the population adopts a MedDiet and experiences 30% reduction in CVD. The pessimistic scenario is a practical short- to medium-term estimate of cost savings that could follow a MedDiet adherence among 15% of the population with 20% reduction in CVD. The very-pessimistic scenario represents a worst-case estimate with only 5% of the population making the dietary change and showing 10% reduction in CVD.

The potential annual economic cost savings for the United States are presented in Table 6. Across the range of MedDiet adoption and CVD reduction scenarios, from ideal to very-pessimistic, the total cost savings were estimated as US $62.8–1.0 billion for 2014 inflation-adjusted dollars, with direct and indirect estimates of US $22.5–0.4 and $40.3–0.7 billion, respectively. In all scenarios, the dollar savings associated with the reduction in CVD-related deaths and the subsequent productivity recoupment (indirect estimates) remained a greater benefit relative to the associated healthcare cost savings (direct estimates). Reduction in prescribed medicine expenditures in particular accounted for nearly half of the direct savings in the total healthcare costs.

**Table 6:** Potential cost savings attributed to the reduction in CVD with varying adoption rates of MedDiet in the United States (US $ billion)

	Scenario^a^			
	
	Ideal	Optimistic	Pessimistic	Very pessimistic
Direct savings				
Hospital, inpatient	5.2	1.3	0.5	0.1
Hospital, emergency	0.5	0.1	0.0	0.0
Hospital, outpatient	2.2	0.6	0.2	0.0
Home healthcare	3.9	1.0	0.4	0.1
Prescribed medicines	10.8	2.7	1.1	0.2
Total direct	22.5	5.6	2.3	0.4
Indirect savings				
Lost productivity/mortality	40.3	10.1	4.0	0.7
Total savings	62.8	15.7	6.3	1.0

a The ideal scenario represents a best-case estimate of potential economic cost savings when 50% of the population follows a MedDiet and shows 60% reduction in cardiovascular disease (CVD). The optimistic scenario is a medium- to short-term pragmatic estimate of potential cost savings when 25% of the population adopts a MedDiet and experiences 30% reduction in CVD. The pessimistic scenario is a practical short- to medium-term estimate of cost savings that could follow a MedDiet adherence among 15% of the population with 20% reduction in CVD. The very-pessimistic scenario represents a worst-case estimate with only 5% of the population making the dietary change and showing 10% reduction in CVD.

## Discussion

Employing data from the established scientific literature and up-to-date monetary figures, this economic analysis assessed the potential annual dollar savings in CVD-related costs within the North American healthcare systems following adherence to a MedDiet. Evidence of considerable opportunities for economic cost savings is provided. Specifically, if between 5 and 50% of the Canadian or American populace follow a MedDiet, an estimated CAD $41.9 million to $2.5 billion in Canada, or US $1.0 to $62.8 billion in the United States, would accrue as total annual savings in direct and indirect costs, given the ‘very-pessimistic’ through ‘ideal’ scenarios. While the former ‘worst-case’ scenario reflects a situation where the general public's MedDiet adoption rate is lowest and poorly translated to beneficial impacts on CVD risk, the latter ‘best-case’ scenario is attainable with higher adoption rates and larger reductions in CVD over the long run. Cost savings associated with the ‘pessimistic’ and ‘optimistic’ scenarios, ranging between CAD $251.1 and $627.8 million in Canada, and US $6.3 and $15.7 billion in the United States, represent short- to medium-term benefits of the MedDiet and are thus realistically viewed as most probable.

The economic cost of disease is generally broken down into direct and indirect components, respectively covering services by the healthcare system and the loss of productivity to society arising from morbidity and premature mortality. The present economic model considered both these components, with some differences between the two countries under study. A particularly important issue regarding international comparability of the study's metrics relates to the differing estimation techniques for productivity effects. These are known to be estimated by one of three approaches. Whereas both the human capital approach and the friction cost approach place monetary value on the productivity costs, the approach recommended by the US Panel on Cost-Effectiveness in Health and Medicine includes part of the value of these costs in the assessment of health outcomes (54). As our data show, the human capital approach implemented by the AHA for the US context, using a 3% discount rate on future earnings, yielded larger estimates relative to the equivalent indirect costs found in Canada, as well as the magnitude relative to direct costs. The indirect costs in the Canadian *EBIC 2005–2008* report implemented the friction cost approach, accounting for surplus employment and the ability to replace a worker. The friction cost approach assumes that after a certain amount of time, referred to as the *friction period*, worker replacement occurs in addition to mechanisms that compensate for the workers absence. Conversely, the human capital approach values mortality and morbidity through decreased productivity during a workers employed lifetime, often valued by a wage rate. Most noted by critics to the human capital approach are the unrealistic assumption of ‘full employment’ and the exclusion of leisure time valuation (55). Pritchard and Sculpher (54) critically review and compare both these approaches, concluding relative merit of each.

In spite of the extensively documented cardio-protective properties of the MedDiet, merely a handful of cost-effectiveness reports have forecasted its potential economic benefits (15). Of those reports, to our knowledge, only one evaluated the healthcare costs associated with a MedDiet, relative to a non-MedDiet intakes, based on estimations of a 10-year risk of developing CHD in 3,042 healthy Greek men and women (56). There, per patient hospitalization costs of €690 were estimated to be the result of a CHD event. Participants who were ‘closer’ to the MedDiet showed a 43% (OR=0.57, 95% CI 0.38–0.86) lower likelihood of having a 10-year risk of over 10%, with a total healthcare cost of €35.880, relative to €336.720 in those who were ‘away’ from the MedDiet. Our observation confirms such an association in the context of a North American setting and calls for an analysis of the outcomes from both the basic research and behavioral nutrition viewpoints.

By design, the conservative calculations presented in this study focused on the cost savings specifically associated with reductions in CVD incidence upon adherence to a MedDiet; however, in reality, other economic benefits are likely to follow higher adoption rates of such a healthy-eating pattern. Such benefits would, for instance, accrue to other agribusiness stakeholders who would respond to a greater demand by the informed general public with a variety of healthy-style plant- and animal-based lines of products that may constitute parts of the typical MedDiet. Furthermore, given that it is an ‘eating habit’ that constellates a number of healthy foods and nutrients, the MedDiet has been shown to lead to improvements in other public health and wellness priorities, such as cancer (57), and is certainly expected to direct similar dollar savings on costs associated with such a major health concern.

The first step towards a MedDiet-associated health and economic benefits is the attainment of a higher adoption rate. This factor is largely influenced by the consumer's food decisions in the marketplace, which are in turn driven by the utility maximization. In economic theory, utility is defined as an unobservable behavioral metric of the satisfaction that the consumers sense with their choice of goods or services. Many factors affect how consumers maximize their utility, or in terms of dietary settings, choose what they eat, including habit, culture, convenience, taste, cost, personal values, and health (58). Research in food marketing also draws attention to a number of the so-called ‘external cues’ that may affect eating habits (59). When consumers are made aware of these factors and sustain behaviors of consuming healthy dietary patterns, health benefits along with concordant economic savings are expected to arise. Specific to the MedDiet, a free-living study promoting higher adherence among a cohort of Canadian participants demonstrated significant reductions in circulating total cholesterol levels and body mass index, two established biomarkers of cardiovascular health, with increased MedDiet adoption rates (26). The scenario analysis of our model sets the groundwork for similar studies in the future, allowing for the computation of economic savings for those wishing to explore the possible avenues and implications of promoting the MedDiet. Dynamics have important implications on the results of this study when considering the adoption of a novel diet. As it cannot be assumed that a MedDiet adoption will be instantaneous, the scenario approach reflects uncertainty in the adoptions of timing and extent. Determining savings across a continuum of adoption paths and CVD reduction rates will provide an opportunity of comparing the costs and benefits of such promotion programs.

The value of dollar savings observed with the MedDiet adoption and CVD risk reduction scenarios in this sensitivity analysis, specifically with the ideal scenario, but also other scenarios, underscores the synergistic responsibilities of stakeholders such as food industrialists and marketers, policy makers, educators, researchers, and healthcare professionals in establishing healthy food environments and attitudes. For instance, creating and advertising lines of healthy food products, appealing to the consumers, by the food industry and marketing sectors should be accompanied by clear guidelines by policy makers and sufficient messages of knowledge by educators and the media to the general public regarding the intrinsic health-related economic importance of those food products. Such action would essentially rely on a body of evidence from well-designed dietary studies and, most important, economic models such as the one of the present study.

This economic model possesses a number of strengths. It is to our knowledge the first to examine the potential economic savings attributed to lower incidence of CVD with adherence to MedDiet in North America. The model template was derived in part from those utilized in Malla et al. (23) and Gyles et al. (24), hence share some similarities. However, the nature of the dietary intervention proposed is distinctly different particularly in that it represents a combination of dietary ingredients in the current analysis compared to single entities in previous research. Investigating whole foods and dietary behaviors in their entirety rather than individual nutrients is logically, and evidently, better in capturing a more realistic view into the potential health and economic benefits for societies. As such, the downstream implications of the dietary transformation are highly disparate for the present MedDiet intervention versus canola and plant sterol interventions. Another strength of the present analysis relates to the joint discussion of Canada and the United States. While it is true that the current dramatic rates of CVD place similar manner of burdens on national resources of the affected societies worldwide, the disease and cost-saving estimates from non-Mediterranean regions hold a special importance. The vast majority of studies associating MedDiet consumption with cardiovascular health benefits emerge from countries of the Mediterranean basin where independent lifestyle factors are likely an influential contributor. This is not the case in countries with the so-called typical North American diet, or Western diet, where other lifestyle factors are unlikely to contribute to a MedDiet intervention's predicted health benefits. The present analysis hence enabled the assessment of benefits of a MedDiet adoption in the absence of contributing ‘healthy’ lifestyle factors that typically accompany this eating pattern in Mediterranean countries. This joint discussion also allowed for establishing a MedDiet's economic benefit in two countries that differ in population size and constructs of healthcare systems; a universal healthcare coverage in Canada and a combination of public and private healthcare efforts in the United States. Additionally, by design, the sensitivity analysis relied on relevant evidence from the established literature and enabled coverage of a range of assumptions for most robust predictions. And, the final economic figures were made possible with employment of the most recent healthcare costs available while adjusting for current monetary values. This model is also subject to some limitations, however. There is no one specific definition for the ‘Mediterranean diet’, given the broad food system and cultural differences in countries where it is typically adopted; ‘Mediterranean-style diet’, as used and defined in this article, is hence a more descriptive term of such an eating pattern. Also, other lifestyle factors in the Mediterranean regions might, as stated previously, have contributed to shaping the reported health benefits of the MedDiet beyond the dietary pattern *per se*. Estimations of the potential adoption and CVD reduction rates in the current analysis were thus based on data from studies in North America and various countries, respectively. In addition, from both the economic and nutrition standpoints, food substitution remains an important issue that was not captured in the analysis, impacting relative quality of health and product prices. It is also important to note that this approach is conservative regarding the responsiveness of hospital-related resource allocation, whereas in the long-term fixed costs are able to adjust. Future research ought to take these points into consideration and include such a dynamic economic construct within the design of carefully controlled human intervention trials of both short and long durations.

## Conclusion

The present novel monetary modeling exercise sheds light on the economic importance of the MedDiet, beyond its well-established health impacts. This may aid in improving policies to increase the consumers’ and policy makers’ general knowledge of the concept of healthy eating, leading to better decisions in the marketplace, and thus improved quality of life. Such dietary guidance directives are expected to ultimately contribute to optimizing the sustainability of healthcare provisions and the handling of national resources as a result of lower social and monetary burdens associated with CVD in Canada, the United States, and possibly other parts of the world.
